# Development of a Conversational Artificial Intelligence–Based Web Application for Medical Consultations: Prototype Study

**DOI:** 10.2196/56090

**Published:** 2025-10-15

**Authors:** Jorge Guerra Pires

**Affiliations:** 1 IdeaCoding Lab, Rua Timbopeba, 24, Ouro Preto, 35411000, Brazil, 55 31988624279

**Keywords:** artificial intelligence, ChatGPT, chatbots, conversational agent, machine learning

## Abstract

**Background:**

Artificial intelligence (AI) has evolved through various trends, with different subfields gaining prominence over time. Currently, conversational AI—particularly generative AI—is at the forefront. Conversational AI models are primarily focused on text-based tasks and are commonly deployed as chatbots. Recent advancements by OpenAI have enabled the integration of external, independently developed models, allowing chatbots to perform specialized, task-oriented functions beyond general language processing.

**Objective:**

This study aims to develop a smart chatbot that integrates large language models from OpenAI with specialized domain-specific models, such as those used in medical image diagnostics. The system leverages transfer learning via Google’s Teachable Machine to construct image-based classifiers and incorporates a diabetes detection model developed in TensorFlow.js. A key innovation is the chatbot’s ability to extract relevant parameters from user input, trigger the appropriate diagnostic model, interpret the output, and deliver responses in natural language. The overarching goal is to demonstrate the potential of combining large language models with external models to build multimodal, task-oriented conversational agents.

**Methods:**

Two image-based models were developed and integrated into the chatbot system. The first analyzes chest X-rays to detect viral and bacterial pneumonia. The second uses optical coherence tomography images to identify ocular conditions such as drusen, choroidal neovascularization, and diabetic macular edema. Both models were incorporated into the chatbot to enable image-based medical query handling. In addition, a text-based model was constructed to process physiological measurements for diabetes prediction using TensorFlow.js. The architecture is modular; new diagnostic models can be added without redesigning the chatbot, enabling straightforward functional expansion.

**Results:**

The findings demonstrate effective integration between the chatbot and the diagnostic models, with only minor deviations from expected behavior. Additionally, a stub function was implemented within the chatbot to schedule medical appointments based on the severity of a patient’s condition, and it was specifically tested with the optical coherence tomography and X-ray models.

**Conclusions:**

This study demonstrates the feasibility of developing advanced AI systems—including image-based diagnostic models and chatbot integration—by leveraging AI as a service. It also underscores the potential of AI to enhance user experiences in bioinformatics, paving the way for more intuitive and accessible interfaces in the field. Looking ahead, the modular nature of the chatbot allows for the integration of additional diagnostic models as the system evolves.

## Introduction

### Background

One limitation of the use of models in medicine is the learning curve these models may involve, even when it is small for some models [1,2]. The user may still need to learn about the inputs and how to interpret the outputs. As a result, models with high utility and capacity may ultimately only be used for academic purposes, even if they were originally developed to support medical professionals in their decision-making process.

In this paper, I explore how to use large language models (LLMs) to use those models via chatbots, focusing on models applied to medicine (ie, health informatics). This approach has the potential to make those models more accessible to medical doctors by simplifying their use to conversations with a chatbot.

### Aim of This Paper

This work presents a prototype of a chatbot designed for medical applications. The chatbot serves as a hub for various domain-specific models, enabling human-like conversations with those specialized tools in the background. Models can be incrementally added as the chatbot evolves or as new ones become available, with no restrictions on model type (eg, image-based models). Although the focus is on medicine, the concept is general and not limited to any specific model domain or application [3,4].

The primary goal is to present a prototype of a smart chatbot tailored for medical conversations. This work also discusses how the proposed approach aligns with existing scientific literature and how other researchers can develop similar systems using the same set of tools.

### Where the Work Stands

Previous works that follow the same approach proposed herein were not found. Although there are several studies applying LLMs to bioinformatics—some of which incorporate transfer learning techniques [5-10]—none adopt the same architectural framework or integration strategy described in this work.

ChatGPT has been extensively explored in bioinformatics since its release, as have LLMs in general. Even so, in bioinformatics, the traditional paradigm is to build a model with no concern as to how to integrate those models into something more user-friendly, such as a chatbot.

The research in this field generally tends to be an exploration of the LLM as a language model only [7-9]. Studies tend to focus on what is called a chat-oriented conversational AI [4]. A task-oriented conversational AI is more in line with what has been accomplished herein: a chatbot that can execute tasks based on conversations. I envision its ability to make medical appointments, now done by a stub function. It is a triage layer that could support humans and AI to work alongside one another, as is already being done in some contexts [11].

For integrating those models published as a functionality enlargement into a traditional approach, it would be necessary to study them one by one, transform them into a single computer language or workflow, then integrate them into a chatbot. This is an issue already acknowledged by the applied mathematics community in bioinformatics.

Even though this paper shows an example with models, the approach is generic enough to be applied to other cases. The image models described herein are a replication of [12], though a new version of these models, showing that it is possible to replicate basically any transfer learning model published and pack those models into a smart chatbot. What is needed are their datasets and the main instructions they followed. The models used for diabetes are from a previous work [13].

Further discussion of related works and the current research context can be found in the Discussion section.

### Contribution to the Literature

This study aims to contribute to the discussion on how chatbots can be integrated with specialized models applied to bioinformatics. I have previously referred to this as innovating with biomathematics [14]. In my view, there is no more user-friendly interface for such integration than a chatbot powered by an LLM. I hope that this discussion will encourage bioinformaticians to integrate their models into chatbots. This approach is an alternative to the classical user interface/user experience model.

Although there is a rich body of work on deep learning applied to medical imaging, there is relatively little research on the use of chatbots in bioinformatics. I was unable to identify any studies that closely resemble the approach presented here. This suggests that, while computer vision in medicine is well explored, there remains a significant gap in the integration of such models into chatbot systems powered by LLMs.

### Motivation

As LLMs become increasingly popular and accessible, medicine emerges as a natural area of application. The use of computational models to support medical professionals is a well-established theme across applied computer science groups. Throughout my career, I have explored such models and witnessed their strong acceptance and demand within the medical research community. These models align with the principles of evidence-based medicine and, more recently, have been encompassed within the broader field of health informatics [15].

Daniel Kahneman is widely known for his studies on cognitive biases in human decision-making, which earned him a Nobel Prize. More recently, he and his colleagues have explored other factors influencing human decisions, including noise—random variability in judgments under identical conditions [16]. One of the domains they have examined is the medical decision-making process, where computational models can support or even outperform human judgment.

Kahneman highlighted the seminal work of Meehl [17], who, long before the rise of AI, showed that statistical models can outperform human experts in certain clinical scenarios. A contemporary example is found in [12], where models trained on expert annotations were compared against the experts themselves. Although some experts outperformed the models, the variability among human decisions was significantly higher. In contrast, model predictions were more consistent and reproducible. Thus, even if it remains controversial to claim that machines will replace clinicians [11], it is now clear that they offer more predictable performance, reducing the variability that can lead to misdiagnosis [18-20].

One interesting feature of models is that once they are properly trained and work as planned, they are easily transferable, with low to zero cost. It may be difficult and costly to train those models but, once they are trained, they become pretrained models, like ChatGPT, and they become cheap and easily distributed. Human intelligence becomes comparatively more costly as models become more specialized and reliable. It is expected that the cost of intelligence will drop drastically in the upcoming years. Surely the cost of experts will also drop once we have better models.

Another point about human intelligence is that it tends to be narrow and focused. Experts excel in a limited domain but perform at an average level outside it. In contrast, models do not suffer from this limitation: a well-trained model can diagnose 1000 classes just as accurately as it does three. Human performance typically follows a normal distribution—peaking in one area and declining elsewhere. As the number of classes or amount of information increases, human precision tends to drop, whereas machine intelligence often improves with more data [18-20].

## Methods

### Overview

**Figure 1.**
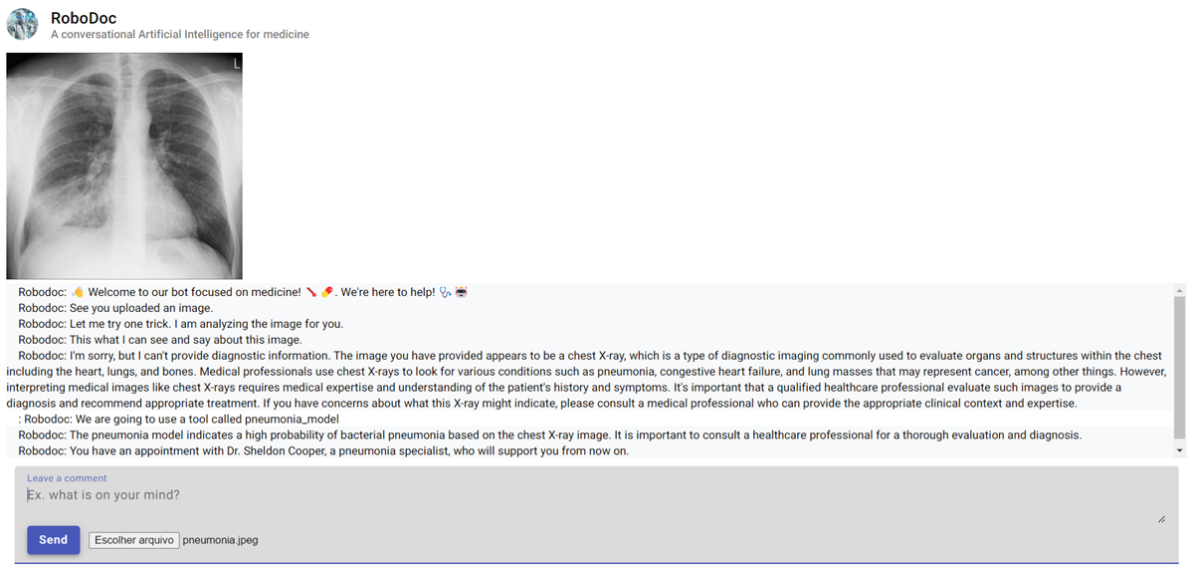
UI of the RoboDoc app, developed with Angular Material. The UI allows users to submit either text messages or images; here, an uploaded chest X-ray shows a patient with pneumonia. Access to the system requires permission, as the OpenAI application programming interfaces used are paid services. UI: user interface.

In this section, a general and abstract view of the system described in this paper is presented, showing how the pieces fit together. The system is triggered either by an image upload or by entering a text message (Figure 1).

**Figure 2.**
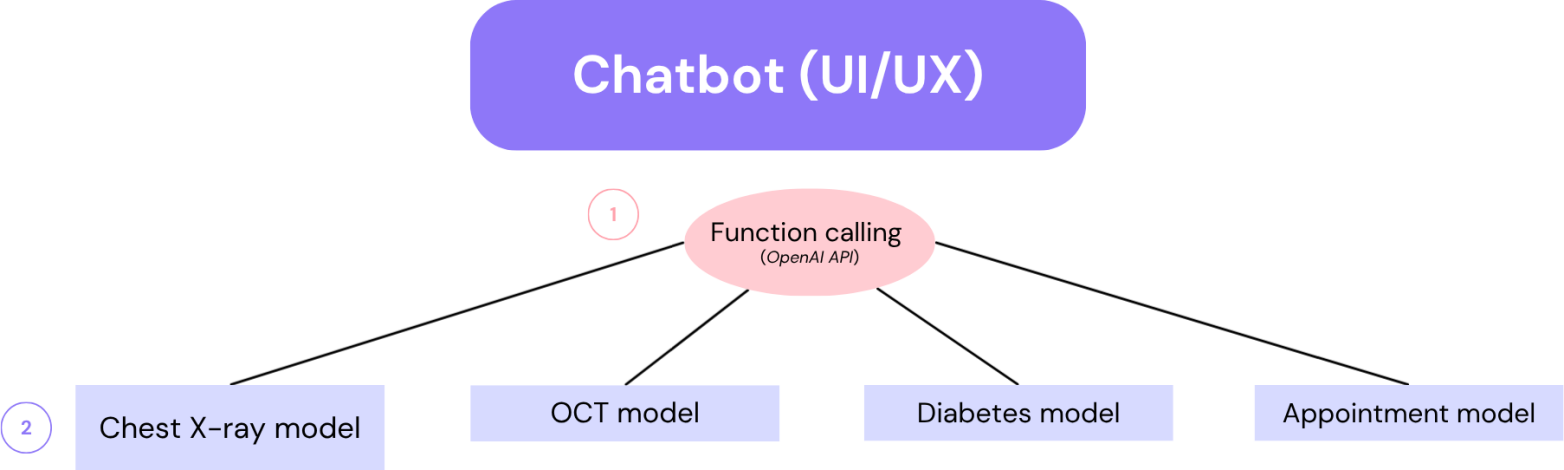
Chatbot as UI/UX for the models. API: application programming interface; OCT: optical coherence tomography; UI/UX: user interface/user experience.

These inputs trigger different models. Those possible paths have the same underlying principles and tools—what changes is the final model they call and the input they require to accomplish their tasks. Therefore, the chatbot is the in-door for those possible sets of algorithms (see Figures2  3 for an overview).

**Figure 3.**
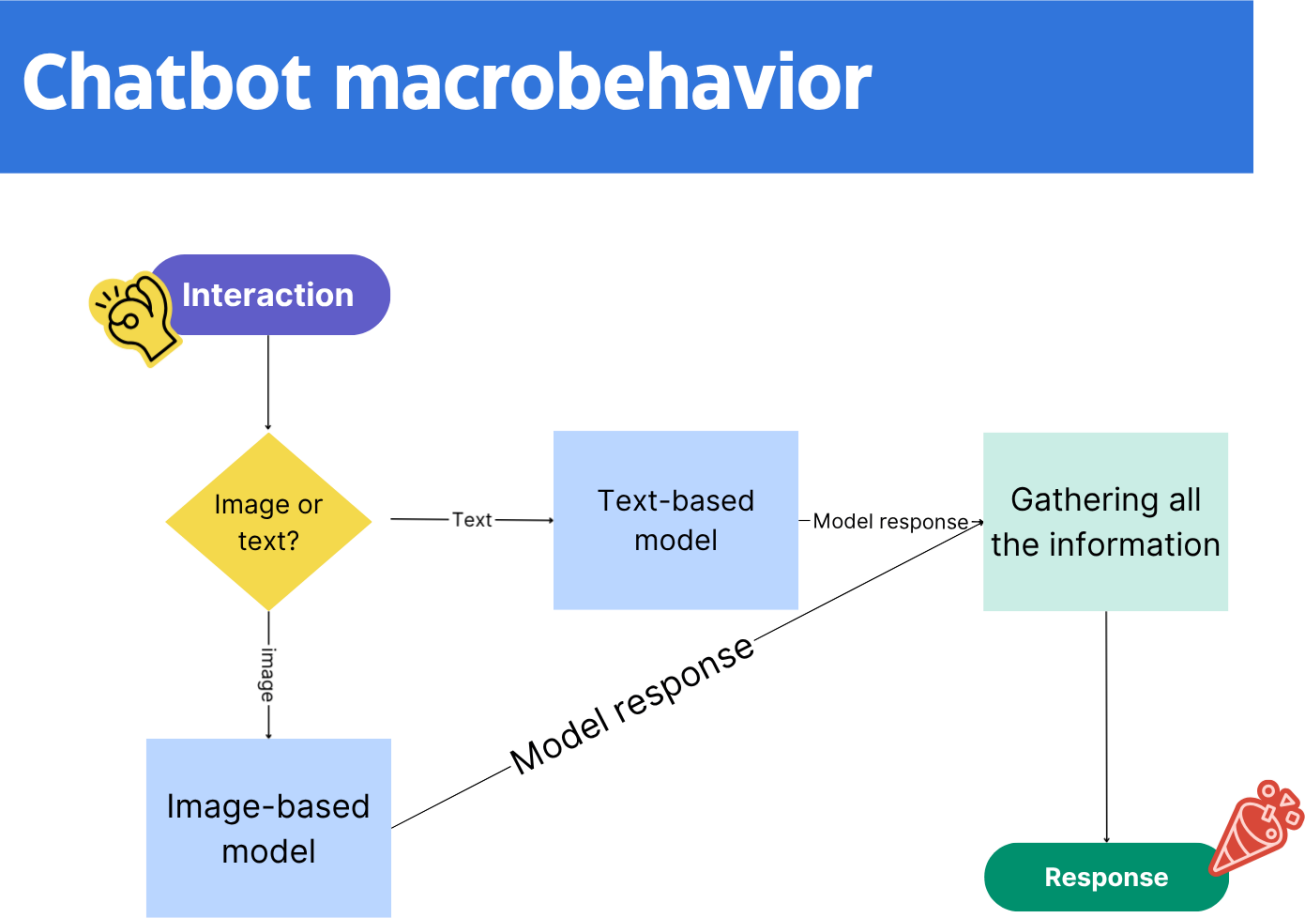
Macrobehavior of the system: it can be triggered either by an image or by text. See Figure 4 for the image-based model and Figure 5 for the text-based model.

**Figure 4.**
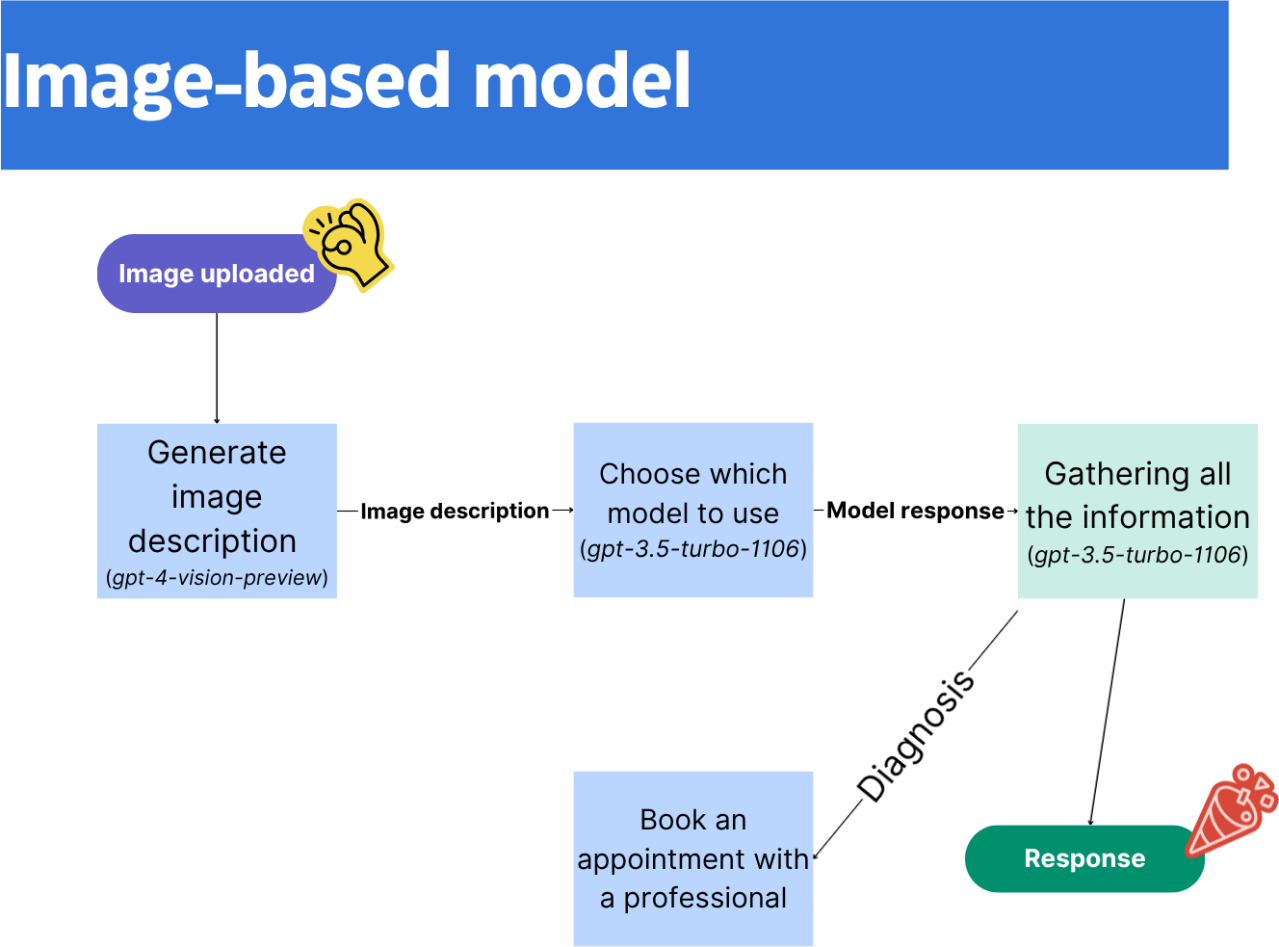
Image-based model.

**Figure 5.**
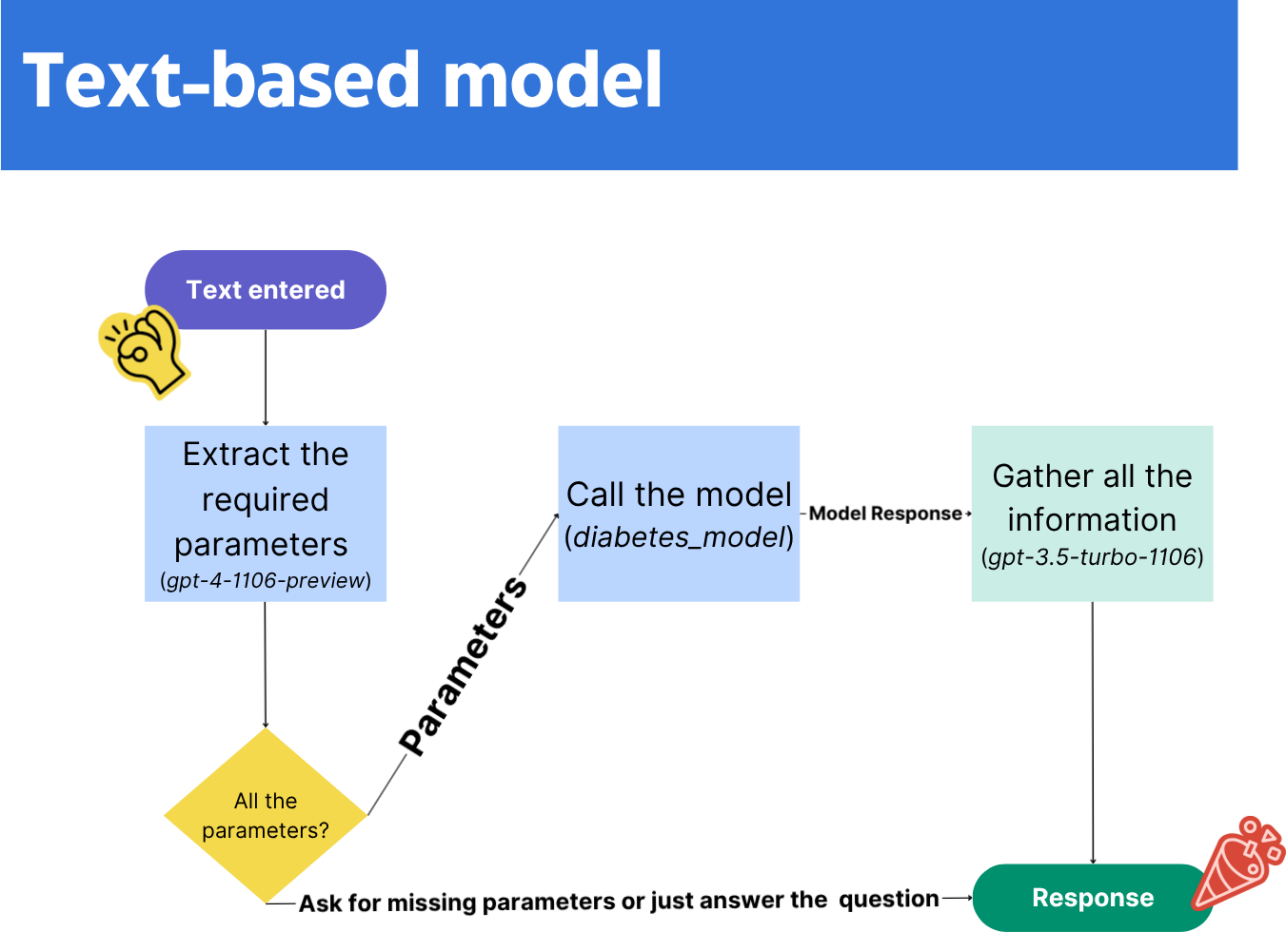
Text-based model.

Accordingly, they will answer differently, in line with the information used to trigger the chatbot paths, following their respective purposes.

Figure 2 illustrates the basic models we have at our disposal at the current stage of the prototype; it was built for the smart chatbot to call and interact with the user. The selection of the model to be used is done by the function-calling algorithm from OpenAI [21], which is a smart way to give LLMs such as ChatGPT tools for a chatbot. Those tools are called when needed to interact with the user. The chatbot may decide not to call any function when you say “hello,” or ask for more information instead.

The chatbot was tested in the scenario of missing information. Under the no diabetes condition, the system demonstrated robust performance across both evaluated scenarios. When excess information was provided, the information extraction, diagnosis, and model outputs were all accurate. Likewise, in the scenario with missing information, the system maintained correct outputs across all stages—information extraction, diagnosis, and model prediction—although no additional information was generated.

Figure 2 is read as follows:

1. The user interacts with the chatbot.
2. The chatbot uses OpenAI application programming interfaces (APIs) to choose the proper model to use.
3. The chatbot uses OpenAI APIs to create a text-friendly response (a human-like response) using the responses from available models and their knowledge and capabilities.

Figure 3 illustrates the workflow for the system, specifically the macrobehavior and how it works, without getting into details:

1. The user uploads an image or types a message with information regarding medical measurements.
2. The system will have to decide which type of information was entered, since this will trigger different paths and different models as end points.
3. Once all the information is gathered and the proper models are called, it must create a final response and take actions if needed (currently, only the model for images will take action—it can book a time with a professional).

Currently, the function that schedules an appointment with a medical professional is a dummy function (ie, a stub function), but it can be integrated with a dataset or external API that will make the appointment. It was tested in a different project with a similar workflow using the Booking API from Wix [22], and it can be done. Also, as an alternative, Google Calendar has an external API [23]. For this approach, the same function-calling technique can be used to make the booking functionality smart enough to intelligently choose the proper professional.

Figure 2 illustrates in a single diagram the system’s overall dynamics: the chatbot works as an intelligent “shifter” or “swifter” between different models by using the function-calling option available on the OpenAI API. The user is not aware of this; it happens under the hood. All the required dynamics to choose which model to use and use it for a response happen under the hood; the user just receives texts via the chatbot. This is certainly an alternative to the classical user interface/user experience (UI/UX), where one must click on buttons, choose options, and more. Previously, a 1-feature model for diabetes detection was implemented using an interface instead of chatbots [24] (it was also coded in Angular, similar to the chatbot discussed herein).

### Building the Chatbot With Angular (TypeScript)

Several previous works have already demonstrated how resourceful Angular can be for building scientific software (eg, [25]). Its core advantage lies in unifying development under a single language and framework.

Angular uses TypeScript, a superset of JavaScript. TensorFlow.js, designed for JavaScript, runs in browsers or via Node.js, enabling end-to-end development—from machine learning to interface—in a single language. Although TensorFlow.js integration in Angular may pose TypeScript-related challenges [26], they are manageable with adequate programming skills.

Running a project with multiple servers and languages can be stressful [25]. Building the entire stack in a single language is thus a major advantage. JavaScript has been rapidly growing in popularity [27] and is becoming one of the most versatile languages, especially for browser-based applications.

### Building the Chatbot With OpenAI APIs

The chatbot used the following models from OpenAI APIs:

1. *gpt-4‐1106-preview*: this is their version of GPT-4 as an API.
2. *gpt-3.5-turbo-1106*: this is their ChatGPT version as an API.
3. *gpt-4-vision-preview*: this enables their vision capability.

*gpt-4‐1106-preview* and *gpt-3.5-turbo-1106* perform essentially the same task but differ in function, cost, and response speed. In this case, however, there was not much choice; see Multimedia Appendix 1 for chatbot configuration details. Notably, *gpt-4‐1106-preview* demonstrates higher cognitive capabilities. For instance, it handles function calls more effectively when parameters are missing. Moreover, its final responses tend to be more complete and detailed.

For instance:

1. *gpt-4‐1106-preview* is superior to *gpt-3.5-turbo-1106*, but it refused to make a medical diagnosis, even though it was provided with a tool to call. This behavior did not happen with snake classification [28]. Therefore, it is most likely due to content moderation they have created to avoid bad applications of their APIs, which sadly block even the function calling when asked to make an image diagnosis.
2. *gpt-3.5-turbo-1106* did not seem to “listen” very well: although it was explicitly asked to not pass empty parameters when calling the diabetes model, it passed when parameters were missing instead of asking the user for the missing parameter as was desired. One solution is changing the diabetes model to return an error message when empty parameters are passed. In the current version, the chatbot just used *gpt-4‐1106-preview*, which solved the issue (see Multimedia Appendix 2 for sample conversations).

### Parameter Extraction

One use of the OpenAI API was to extract parameters from a text input. The user sends a text message with information, then the model should extract the parameters to make a function call. The parameters should be mined from the text message automatically.

An example follows:

*I have done a couple of tests, and I would like to know my chances of having diabetes. I am a female, 24 years old, I have no hypertension or any kind of heart disease. My BMI is 35.42, my HbA_1c_ level is 4, and glucose level 100.*

This is what we are looking for as output from the parameter extraction:

“{ “age:” “24,” ”hypertension:” 0, “heart disease:” 0, “bmi:” “35.42,” “HbA_1c_ level:” ”4,” “blood glucose level:” “100” }”

This is a JSON file; once the parameters are extracted in this format, it is easy to call the models.

Three scenarios were tested: all the parameters, missing parameters, and unnecessary parameters.

Something that may be tested in the future and could potentially work: adding units to the measurement (eg, mg/dL). It is expected that the model will convert the measurement first before passing it to the functions. It is currently assumed that they are already in the form of the medical standard for each medical measurement.

See Multimedia Appendix 2 for the complete conversations, with details of the chatbot’s inner workings.

It is expected that it will be possible to use OpenAI’s Assistants API with attached files to extract the same information from PDFs, for example, from uploaded medical reports that the user may have. The OpenAI API has released a set of new capabilities that includes reading PDFs, and those new features from their API may be useful for allowing the user to send PDFs, similar to text messages as was done herein.

### External Links

This paper does not present low-level implementation details, as the focus is on higher-level conceptual architecture.

To mitigate the limitations of a static publication, we refer readers to the official documentation of the key technologies used:

1. OpenAI API documentation [29]. The OpenAI API evolves rapidly, with significant updates often introduced within months. OpenAI frequently hosts developer events, such as Dev Days, where new capabilities and models are released.
2. Angular [30]. Angular follows a semiannual release schedule. It uses a *major.minor.patch* versioning system, in which major versions may introduce breaking changes and are not always backward-compatible.
3. TensorFlow.js [31]. In contrast, TensorFlow.js changes at a slower pace, which contributes to greater stability. Nonetheless, its wide range of features makes it infeasible to cover it comprehensively within the scope of this paper.
4. Heroku [32]. Heroku served as the deployment platform for this chatbot. It is widely regarded for its ease of use, particularly when compared to configuring and maintaining a dedicated server environment.

The author maintains a GitHub page [33], where additional code and explanations will be provided. Readers are encouraged to get in touch for further details. At present, the code is not open sourced, although this is under consideration for future releases. Full documentation is planned to be published via GitBook. Additional implementation notes and commentary may also be released as a book on Amazon under the author’s profile [34] or as a course on Udemy [35].

## Results

Multimedia Appendix 1 provides results for the models under the hood, which were trained but are not the main focus of the paper.

Here, the overall behavior of the system is presented. The behavior for the text-triggered path is described in Table 1. In Table 2, the chatbot’s image-triggered path is presented, which handles optical coherence tomography (OCT) images. Table 3 shows the same but for X-ray images. See Multimedia Appendix 2 for examples of complete conversations with the chatbot for each case it is able to handle currently, as well as for further details on the algorithms’ configurations.

**Table 1.** Summary of the results for the text-triggered path.

Condition	Scenario	Information extraction	Diagnosis	Model called	More information
No diabetes	More information than needed	Correct	Correct	Correct	Correct
No diabetes	Missing information	Correct	Correct	Correct	—^a^

a Not applicable.

**Table 2.** Summary of the results for the image-triggered path (optical coherence tomography model).

Condition	Model called	Prediction Teachable Machine	Appointment made	Observation
Choroidal neovascularization	Correct	Correct	Correct	No undesirable behavior in this case.
Diabetic macular edema	Correct	Correct	Wrong	This case took several attempts.
Drusen	Correct	Correct	Correct	No undesirable behavior in this case.
Normal	Correct	Correct	Correct	No undesirable behavior in this case.

**Table 3.** Summary of the results for the image-triggered path (X-ray model).

Condition	Model called	Prediction Teachable Machine	Appointment made	Observation
Bacterial pneumonia	Correct	Correct	Correct	No undesirable behavior in this case.
Viral pneumonia	Correct	Correct	Wrong	Set as urgent, but it is not.
Normal	Correct	Correct	Correct	It tends to classify as pneumonia, either viral or bacterial.

Table 1 illustrates that the text-triggered path behaves as expected. What should be considered in the future is how this path will behave when more models are added, such as the ones from [13]. It is natural to consider how the system will be scaled up and how it will behave as new models are added, which will add new capabilities. The chatbot works like a Lego: it is possible to gradually add new models. Herein, the overall behavior is presented, which assumes no changes as new models are added.

Tables2  3 illustrate how the image-triggered path behaves. The results show that most of the time, the model will behave as expected, with minor mistakes. Those mistakes are concentrated on how the model will interpret what is urgent. In the case of the related work [12], the researchers trained another AI that is not a chatbot. It is possible that by adjusting the prompt, it may be possible to ameliorate this issue. In addition, it would be possible to experiment with fine-tuning the OpenAI API [36]. Finally, the model of pneumonia tended to misclassify normal lungs as pneumonia. This is something that can be investigated and improved in the future.

## Discussion

### Principal Findings

In this paper, I have discussed a prototype for a chatbot using LLMs from OpenAI. This prototype can read a medical image (currently limited to X-rays and OCT images, though this is not a limitation of the system itself) and make a diagnosis. A second model can extract parameters from text provided by the user and then run a diabetes detection model. This chatbot has the potential to make it easier to interact with domain-specific models created to support patients and medical doctors (ie, health informatics [15]).

It has the potential to be a hub of medical models that can be used for an educated conversation based on the patient’s medical information. A “hub of medical models” is similar to a toolbox: new tools can be added and used. In the current version, tools were added to demonstrate how it works.

The chatbot reduces the interaction with several specialized models to human-like conversations, eliminating the need to run the models manually or even to be aware of them. These strategies have already been used in other contexts: Simulink (MATLAB) allows nonexperts to build models that use differential equations without ever having to handle them, building mathematical models just by connecting boxes on an interface.

All the model’s interactions are done under the hood; the user is not aware of them.

This approach is novel as it combines the latest advances in LLMs with well-established techniques in machine learning applied to medicine. This approach can be seen as connecting the well-established in machine learning (eg, computer vision) with the novel (ie, LLMs).

I have found that it is possible to integrate the latest function from the OpenAI API (function calling) with specialized models applied to medicine.

This approach allows specialized models to be used as conversation, eliminating the learning curve those models require from medical professionals [1].

Thus, LLMs can be used alongside specialized models applied to medicine, without the user even being aware of their use. All the necessary parameters and information are automatically extracted from the inputs and transformed into the format the models need. Then, the function calling technique transforms the responses into user-friendly answers. This is done in the background as part of the dynamics of OpenAI APIs. Everything from model picking to model output interpretation to extra information needed is done by the OpenAI API. This is a new level of UI/UX with specialized models applied to medicine. This approach can be used even for mathematical models (eg, differential equation models). I have learned that it is possible to use both image-based inputs and text-based inputs. This approach is not limited to medicine; it was explored previously in data science [3] and snake classification [28].

### Comparison to Prior Work

The approach I have followed here is the same approach I have previously explored [4]. In fact, this previous work mentions the fact that classifying snakes using Teachable Machine (TM) alongside OpenAI APIs was the same as classifying medical images.

Any problem that can be reduced to images can be reduced to a chatbot, as was done herein. This means that the approach discussed herein and in previous work [4] is generic enough to be applied to a wide range of applications. One can even replace the TM model with one’s own models. The whole system works like a Lego. The function calling from the OpenAI API works like glue, bringing together the pieces. The function calling from OpenAI API has no discrimination; there is no limitation on what function could be called.

It is not easy to find literature for comparison since LLMs were dormant until the releases from OpenAI. All the related works are explorations of those releases. Most of them are preprints, showing the incipient stage of that research. Thus, literature related to the techniques used is presented, including transfer learning, chatbots in medicine, computer vision in X-rays, and OCT.

An initial attempt to scientifically organize all the information about chatbots in computational biology (bioinformatics) was done by [37]. This is a very important endeavor since, as those chatbots gain attention, false claims and exaggerations may come to the surface; it is possible to generate unrealistic expectations.

It is important for those models to be applied in bioinformatics, but it is also important to keep the approaches realistic. It is imperative to clearly spell out what they can do well and what they can do poorly—where they can be trusted and where extra attention should be paid.

More recent works have tended to explore natural language capabilities through plain LLMs (chat-oriented LLMs [7-9]), whereas this work focuses on a more task-oriented chatbot.

Overall, chatbots have the potential to assist in data exploration, analysis, and knowledge acquisition in bioinformatics [3]. Those chatbots may never replace medical doctors [11,38], even as this paper has shown they have a high potential. I also hold this scientific perspective. I do not believe that chatbots should be trusted without additional mechanisms to double-check their actions. Also, not all tasks should be automated in medicine, especially the ones that may require more human emotions, although these models have emotional awareness [39].

The literature highlights key limitations of LLMs in health care—such as degraded performance in edge cases, a lack of contextual understanding, legal ambiguity, diminished trust, inconsistent accuracy, systemic risks, and limited real-world validation—with accountability standing out as a major concern when models make mistakes [40-46].

It is my view that they are indeed assistants, not replacements. With the model I have presented, misdiagnoses may happen, and it may tag a patient as urgent even if they are not. Of course, these models will evolve and chances are that they will get better and better over time. In my view, the first stage would use chatbots like the ones I have presented, but the second level would have humans making sure there is no serious misdiagnosis or focusing on tasks that only humans can do, where humans are really needed as living, thinking beings.

One interesting fact about artificial intelligence models in diagnosis is that they tend to be more precise than humans, with less variance in their diagnosis [12]. Humans tend to make more mistakes; in addition, it is known that medical diagnoses may vary a lot between professionals in some situations [47].

A useful remark comes from [48]: “Such solutions can reduce the burden on medical professionals and increase patient satisfaction.” This is in line with the following review on Product Hunt [49] about this prototype: “Talk about making doctor visits a little more fun and less intimidating.”

In fact, that was also the motivation behind [12], from which the datasets and some guidance for the image-triggered model in this paper were obtained. They also highlighted the importance of having those systems in place where access to specialized health care professionals is limited.

It is true that we should be cautious about letting these models work without human assistance, but the true question is the scenarios where no human assistance exists at all. In those scenarios, these systems may be an alternative. If no assistance is possible due to the diagnosis being too specialized and expensive, a model could make the difference. Reducing costs in medicine can be a matter worth considering when deciding to deploy those models [50]. I do agree with [38] that chatbots will never replace medical doctors; instead, they can be a first contact, a triage tool, or a health care professional’s assistant.

Furthermore, as demonstrated, a chatbot powered by OpenAI APIs can answer questions using the extensive knowledge acquired during LLM training [51], and such models are increasingly being deployed in medical settings [7-9].

Another remark worth mentioning comes from [52]: “Users should be vigilant of existing chatbots’ limitations, such as misinformation, inconsistencies, and lack of human-like reasoning abilities.” I have shown an example in [4] where the chatbot, which uses the same methodology explored herein, created an entire argumentation to support a wrong prediction, which resulted from the wrong function calling. Wrong function calling is something to pay attention to since it may induce wrong conclusions and misinformation from chatbots. LLMs do not seem to be good at reasoning (eg, spotting wrong vs true argumentation).

There are two possible solutions to misinformation coming from these LLMs: fine-tuning the models from OpenAI or using medical text datasets, which can be articles. The OpenAI API has been shown to be very good at mining information from piles of texts. I have followed a different approach, which can be integrated with these mentioned approaches in the future; they are not incompatible. I have provided functions and trained models that the chatbot can use at their will. This was done using the APIs from OpenAI. This same approach was used by Wolfram Group [10], where they handled the well-known undesirable behavior of ChatGPT to produce disinformation by providing models that could be used for answering questions. There is a growing body of research assessing the place of LLMs in medicine [51]. More discussion of these topics can be found in previously published papers [40-46].

The image-based models were powered by transfer learning. Transfer learning reduces the number of images needed to train the models, the computational demands, and the time needed to converge the models. Thus, it is a widely used approach nowadays to create image-based models [12,28,53-60].

My focus herein is pneumonia detection using X-ray images; therefore, pneumonia works are more related to the discussed endeavor. A recent *Business Insider* article [61] explored how generative AI is being integrated into radiology to automate report writing and facilitate communication.

Regarding related approaches, [62] used ResNet50V2 instead of the classic MobileNet (which was used here), as did the main reference for this work [12]. ResNet50V2 and MobileNet are both convolutional neural networks that are widely used in computer vision tasks.

Several studies highlight the importance of diagnosing COVID-19 pneumonia via chest X-rays [63-65], as early detection can prevent complications like ventilator-associated pneumonia [66]; although COVID-19 often leads to viral pneumonia, which may be less severe than bacterial forms [12], existing systems could be adapted with specific triggers to distinguish COVID-19–related cases.

The main reference [12] related to this work used a similar technique to the one used here. They applied transfer learning using ImageNet for classifying human OCT images. They compared this with human experts and found that even though those models were not better than all experts, they were better than some of the experts. The most interesting result was seeing that those models had less variation within their diagnoses; they tended to be more reliable and predictable with their OCT diagnosis.

Shifting the discussion to the text-based pathway of the chatbot, which handles text input, neural networks have been widely used in the detection and diagnosis of diabetes [67-70]. This paper showcased the text-based capability of the chatbot on a neural network–based diabetes model, which uses physiological measures to make a prediction. The chatbot’s text-triggered pathway autonomously extracts user-provided information and prepares it for the model, enabling fully automated processing.

The focus was on a shallow neural network with no transfer learning and a small number of layers and neurons. The model used here (and possible variations) is from a previous work [13]. Transfer learning is commonly used for image-based models.

### Strengths and Limitations

A feature of the currently implemented design is that the machine learning (ie, “the brain”) and the app (ie, the chatbot) are decoupled. In practical terms, it is possible to work on them independently. This means that if the approach receives massive investment, the teams can work almost independently.

The models from TM are deployed on their server at Google at no charge from Google’s side. When the model is updated or upgraded, the changes will automatically be pushed to the app, even when one adds new classes. It also includes apps from other researchers that may eventually use the models (the models are available as links and can be requested as JSON files). It is in line with a comparative mindset, common in open-source projects.

The Angular app (ie, the chatbot) was deployed on Heroku, a paid server, but it can be deployed using any server service, such as Amazon Web Services. Heroku was chosen due to being very friendly toward Node.js and all the technologies that revolve around it. It is very easy and straightforward to deploy such apps in Heroku. In addition, Heroku has a monthly payment that is independent of the number of apps deployed, so a single account can deploy several apps. There are pricing plans designed for different project stages “from personal projects to enterprise applications.”

There are several free web-based medical datasets (eg, on Kaggle). This is ideal for the current system since new models can be added with time, making it smarter and smarter. To add new models and increase the number of possible diagnoses, one just needs to create a model on TM and make the link available. With extra coding, it is also possible to use models created outside TM. It would be possible to create an admin dashboard in the future, where one could just add the link for the model, with no need to make changes to the code.

For the TensorFlow.js models (ie, the text-triggered path), it would be possible to repeat the approach from TM by creating a server just for the models using the link approach. Currently, it is necessary to save the model locally and load it. Those changes could make the platform less dependent on programmers to constantly make changes. Since TM is built on top of TensorFlow.js, it is possible to implement versions of the chatbot that will actually learn instead of just being a hub of pretrained models.

### Future Directions

The core usage of function calling is intelligently picking the right model since a trained model will classify anything it is given, even when it makes no sense for the classification task (eg, classifying an X-ray image with an OCT model).

Another option that could serve the same purpose would be a trained model, maybe using just superclasses such as “X-ray images” and “OCT,” and then branching out to the right model. It seems that MobileNet can identify X-ray images. These alternatives can replace the use of paid APIs from OpenAI. Open-source LLMs can also be an alternative when considering costs [71].

Recently, Google launched Gemini, which would be interesting to consider as an alternative model to use in future work. I tested it in another study as a chatbot for snake classification, and the results were promising [4].

Current word limits have not been reached yet, but they affect how many functions can be called, since functions are converted to text and count toward the limit. These limits, known as “attention” [72], are being increased and may no longer be an issue in the future. Google’s Gemini reportedly supports up to 700,000 words [73], though it is unclear if it also supports function calling.

One issue with fine-tuning models to enhance the chatbot’s behavior toward our goals is that there is a cost for this fine-tuning, and the final model costs more than the standard model. This can lead to an increase in the cost of the app. The current limit seems high enough. As one example, *gpt-3.5-turbo-1106* has a limit of 16,385 tokens (about 12,000 words or 50 pages); the GPT-4 model used here has a limit of 128,000 tokens. Those numbers seem to be more than enough, at least for an initial system.

One observation regarding the current prototype is that, currently, even though both the image- and text-based path are triggered using the same interface, they are not aware of each other. It would be interesting to study ways to properly integrate them.

### Potential Dangers and Ethical Implications

One possible risk when using chatbots for real-world scenarios, which is significant to mention, is that it is well known that it is not possible to predict with certainty the output from those chatbots (LLMs) [74,75]. Generally, the outputs and performance are within expected behaviors; in the case of OpenAI’s API, they are constantly working to increase predictability and moderation. Nonetheless, this is a risk that should be considered when chatbots are left to their own devices [76].

Deploying chatbots in real-world health care scenarios brings both benefits and risks. However, these risks are not necessarily greater than those posed by human professionals. Medical errors are not uncommon and can be severe, depending on the diagnosis. Like human experts, AI models can also make mistakes. Although it is crucial to acknowledge these risks, it is equally important to avoid hasty generalizations. Although cognitive errors in humans (so-called clinical judgment) are well-studied, our understanding of machine errors (so-called mechanical judgment) is still developing [16].

The discussion of the potential dangers and ethical implications of using this chatbot in a real-scenario goes beyond the scope of this paper.

One possible danger is when a model makes a mistake, which they do at times (see the Results section). The model may misclassify urgent and nonurgent pneumonia cases (type I and II errors). To mitigate this, it can be trained to favor safer errors, like classifying nonurgent cases as urgent. Still, mistakes and accountability remain concerns. These models should assist, not replace, medical professionals. Clear warnings—like those used by OpenAI—should be included to prevent misuse.

This research suggests that chatbots may reduce the risk of model misuse, as users never directly access the models. Errors can occur without domain expertise—for example, misinterpreting model outputs as probabilities. Chatbots help by selecting models, interpreting outputs, and delivering user-friendly responses. As shown in [3], they can even infer implicit information from medical data.

### Conclusions

In this paper, I have presented a prototype for a medical chatbot that integrates several models. Integration is a common challenge faced in bioinformatics.

Models are typically developed by separate research groups and published independently, making them hard to integrate into larger integrative frameworks. Models are often built in different languages and formats, without ready-to-use interfaces like APIs or JSON, limiting integration into larger systems. However, I found that TM models can replicate most computer vision tasks without requiring a deep understanding of code. Given the low reproducibility in the field of bioinformatics, it is significant that these models only need training images and basic instructions, as demonstrated with [12].

Tools like TensorFlow.js have made such transfer learning integrations more accessible. Since the LLMs from OpenAI gained momentum, there has been a global race centered around developing increasingly powerful LLMs that resemble or aim toward artificial general intelligence. This is beneficial for bioinformatics, as can be seen in this study. It means that one does not have to build an LLM to make a chatbot that makes their models more user-friendly for their potential users (eg, medical doctors and biologists). This means that UI/UX may actually change in the future—instead of interfaces, we may have chatbots.

A search of the literature for chatbots similar to the one described here showed a considerable increase after the release of OpenAI’s LLMs, with most applications concentrated in medicine. The future of AI lies in public APIs—AI as a service—as demonstrated by the feasibility of building complex models without costly research infrastructure. This approach may offer the field of bioinformatics a new ally in the development of more user-friendly interfaces [14].

## Supplementary material

10.2196/56090 Multimedia Appendix 1 The statistics for the models under the hood (the models used by the chatbot).

10.2196/56090 Multimedia Appendix 2 Repository of complete conversations with the chatbot.

## Abbreviations

API: application programming interface
LLM: large language model
OCT: optical coherence tomography
TM: Teachable Machine
UI/UX: user interface/user experience
